# Heterogeneity of Persistence of *Salmonella enterica* Serotype Senftenberg Strains Could Explain the Emergence of this Serotype in Poultry Flocks

**DOI:** 10.1371/journal.pone.0035782

**Published:** 2012-04-24

**Authors:** Zineb Boumart, Sylvie M. Roche, Françoise Lalande, Isabelle Virlogeux-Payant, Christelle Hennequet-Antier, Pierrette Menanteau, Irène Gabriel, François-Xavier Weill, Philippe Velge, Marianne Chemaly

**Affiliations:** 1 INRA, UR1282 Infectiologie Animale et Santé Publique, Nouzilly France; 2 ANSES, Laboratoire de Ploufragan-Plouzané, Unité Hygiène et Qualité des Produits Avicoles et Porcins, Ploufragan, France; 3 INRA, UR83 Recherches Avicoles, Nouzilly, France; 4 Institut Pasteur, Unité des Bactéries Pathogènes Entériques, Centre National de Référence des Salmonella, Paris, France; 5 IFR136 Agents transmissibles et Infectiologie, Université François Rabelais de Tours, Tours, France; East Carolina University School of Medicine, United States of America

## Abstract

*Salmonella enterica* serotype Senftenberg (*S.* Senftenberg) has recently become more frequent in poultry flocks. Moreover some strains have been implicated in severe clinical cases. To explain the causes of this emergence in farm animals, 134 *S.* Senftenberg isolates from hatcheries, poultry farms and human clinical cases were analyzed. Persistent and non-persistent strains were identified in chicks. The non-persistent strains disappeared from ceca a few weeks post inoculation. This lack of persistence could be related to the disappearance of this serotype from poultry farms in the past. In contrast, persistent *S.* Senftenberg strains induced an intestinal asymptomatic carrier state in chicks similar to *S.* Enteritidis, but a weaker systemic infection than *S.* Enteritidis in chicks and mice. An in vitro analysis showed that the low infectivity of *S.* Senftenberg is in part related to its low capacity to invade enterocytes and thus to translocate the intestinal barrier. The higher capacity of persistent than non-persistent strains to colonize and persist in the ceca of chickens could explain the increased persistence of *S.* Senftenberg in poultry flocks. This trait might thus present a human health risk as these bacteria could be present in animals before slaughter and during food processing.

## Introduction

*Salmonella* spp. are among the main causes of food-borne bacterial gastroenteritis in industrial countries. The serotypes most frequently associated with human illness are Enteritidis and Typhimurium, mainly due to the consumption of contaminated chicken eggs and meat [1]–[3]. Although the prevalence of these serotypes has fallen over the last decade in Europe, the emergence of some other *Salmonella* serotypes has been observed at the same time. For example, in recent years the prevalence of *Salmonella* enterica serotype Infantis among poultry and humans has increased [4], [5]. This is not yet the case with *Salmonella* enterica serotype Senftenberg (*S.* Senftenberg), but the recent emergence of this serotype in poultry flocks is a cause of concern.

In poultry production in the past, *S.* Senftenberg was frequently isolated from hatcheries but gradually disappeared during animal rearing [6]. On the poultry farm, development of complex digestive microbiota [7] and maturation of the immune system, allowed *S.* Senftenberg strains to be eliminated from infected chicks. However, the traits of this serotype have changed and it has now become more frequent in animals and on poultry farms. Some strains are able to persist throughout the rearing period, thus representing a potential risk for consumers [8]–[10]. This health hazard is strengthened by the existence of several intestinal and hospital-borne infections in humans linked to *S.* Senftenberg [11], [12].

The aim of our study was to investigate the existence of strains with different levels of persistence in chickens which could explain the increased presence of this serotype in animals and on poultry farms, and to determine whether some strains represent a potential risk to human health. Avian isolates of *S.* Senftenberg from hatcheries and poultry farms, together with human strains were collected, and the genetic relationship between these isolates was assessed using Pulsed-Field Gel Electrophoresis (PFGE). Based on this analysis, some isolates were selected to investigate their ability to persist and colonize chickens, invade avian and human cells and induce systemic infection in mice.

## Materials and Methods

### Bacterial strains

Avian *S.* Senftenberg strains (n = 105) were obtained from monitoring programs, prevalence surveys and private laboratories. Human *S.* Senftenberg strains (n = 29) were provided by the French National Reference Centre for *Salmonella*, Institut Pasteur, Paris.. All isolates were collected from independent sources at different dates. Possible redundant strains were discarded. The *S.* Enteritidis LA5 wild-type strain is a field isolate from infected chickens [13].

### Genetic analysis

Pulsed-Field Gel Electrophoresis of 134 isolates of *S.* Senftenberg was performed using the XbaI restriction enzyme following the procedure developed by the Centre for Disease Control (CDC) [14] and as previously described [15]. The Simpson index of diversity was used to calculate the ability of the method to discriminate between patterns [16].

Multi-Locus Sequence Typing (MLST) was performed on a subset of 23 selected from the 134 isolates, as previously described [17]. Data were submitted to the MLST database website (http://mlst.ucc.ie/mlst/dbs/Senterica).

### Animal experiments

Based on our previous genetic study, 12 strains of *S.* Senftenberg were selected to check their ability to colonize broiler chickens. Spontaneous rifampicin-resistant strains of *S.* Senftenberg (100 ug/ml) were used to facilitate the detection of the inoculated strains. To validate the use of these strains, we demonstrated that the acquisition of rifampicin resistance did not modify the virulence level of the strains. One-day-old chicks (JA957 line) were orally infected with 10^5^ colony-forming units (CFU) of *S.* Senftenberg and 15 chicks were sacrificed every week for five weeks post-infection (p.i). Ceca were removed, homogenized in buffered peptone water, serially diluted, plated on Brilliant Green agar (BG) with rifampicin and incubated at 37°C. In the absence of growth, samples were enriched in Modified Semi-solid Rappaport Vassiliadis medium (MSRV) at 41.5°C for 24–48 h and plated on BG agar with rifampicin.

In a second set of experiments, two persistent (SS308, SS209) and two non-persistent (SS304, SS278) strains were selected from the 12 strains and used to inoculate chicks orally. Spleens and ceca were removed aseptically every week p.i. One cecum was used to determine the bacterial level and the second for histological analysis. Sera were taken from 10 infected and 10 uninfected animals every week.

The virulence potential of *Salmonella* strains was analyzed in the mouse model. Six- to eight- week-old female BALB/c mice (Janvier, France) were orally inoculated with 5×10^8^ CFU of persistent (SS209, SS308), non-persistent (SS278, SS304) and human (SS291) *S.* Senftenberg strains, or the *S.* Enteritidis LA5 strain, as previously described [18]. At three days p.i, the ileum, colon, mesenteric lymph nodes (MLN) and spleen were aseptically removed, homogenized in TSB, serially diluted and plated on *Salmonella-Shigella* agar (37°C, 24 h). Homogenates were kept overnight at 37°C for enrichment. For lethality experiments, survival was recorded twice a day and at 28 days p.i, the ileum, colon, MLN and spleen of surviving animals were removed, and bacterial colonization was assessed. Animal experiments were carried out in strict accordance with French recommendations. The protocol for the study using chicks and mice was approved by the ComEth Afssa/ENVA/UPEC (10-0032) and the Val de Loire (N 2011/10) Ethics Committee for Animal Experiments respectively.

### Histological analyses

Samples of cecal tissue in the proximal region (5 mm) were taken from six animals per group and analyzed according to microdissecting procedures, as previously described [19]. The heights of 10 villi and the depths of 20 crypts were measured in six animals per group using an optical microscope and viewer analysis software (visilog 6.3, Noesis), as previously described [20]

### Quantification of the humoral immune response of chicks

Serum samples from infected and healthy animals were taken every week to detect immune response markers (IgM and IgY) using an ELISA assay, performed as previously described with some modifications [21]. The 96-well ELISA plates were coated with 100 µl of bacterial suspension (10^7^ bacteria/well) containing the four heat-inactivated *S.* Senftenberg strains (SS278, SS304, SS209, SS308). Samples were diluted and incubated in secondary antibodies, either peroxidase-conjugated Goat anti-chicken IgM or Rabbit anti-chicken IgY, and revealed using tetramethylbenzide (TMB) according to the supplier's instructions (Uptima, France). All the experiments were performed in duplicate.

### Invasion and multiplication assays

Human adenocarcinoma HT29 (ECACC n°85061109), chicken hepatocellular carcinoma LMH [22] and avian HD11 and murine J774 macrophages (ECACC n°85011428) were maintained under the conditions described previously [23], [24]. Prior to infection, macrophages were activated with LPS (0.05 µg/ml) in the medium without antibiotics. Bacterial invasion and multiplication were quantified using a gentamicin protection assay, as previously described [25].

### Statistical analyses

Data from the mice experiments were analyzed using the Systat 9.0 software (SPSS Inc., USA). Differences between means were determined using a one-way analysis of variance (ANOVA) followed by a student *t*-test. Values of *P*≤0.05 were considered significant.

Data from the chicken experiments were analyzed using proc glm in SAS®. For cecal colonization, we performed an analysis of variance to study the response log CFU/g in terms of the effects of day, strain and day*strain interaction. To identify persistent from non-persistent strains, level of the day factor was determined from 19 days to 26 or 33 days p.i. The effects (day, strain and day*strain) are significantly different from zero at the 0.05 level. Pairwise comparisons of the day *strain effect were carried out and the p-values of hypothesis tests were adjusted using the Tukey method. The strains are clustered from the distance matrix calculated by 1 - p-values and a complete linkage as agglomerative algorithm. The heatmap and dendrogram summarize the results of the comparison tests of the means of the strains.

An ANOVA was performed on the results of spleen colonization to study the response log CFU/g in terms of the effects of day, strain and day*strain interaction. When the interaction day*strain was significant, the pairwise comparison was performed. In addition, we were interested by the contrast between persistent (SS308, SS209) and non-persistent (SS304, SS278) strains. Histological analysis data were analysed using Stat-View 5.0 software (Abacus concepts, Berkeley, CA, USA). Differences between the means of villus heights or crypt depths were determined using the Kruskal-Wallis test followed by the Mann-Whitney test at a 5% level of significance (*P*≤0.05).

## Results

### Genetic analysis of *S.* Senftenberg isolates

**Figure 1:**
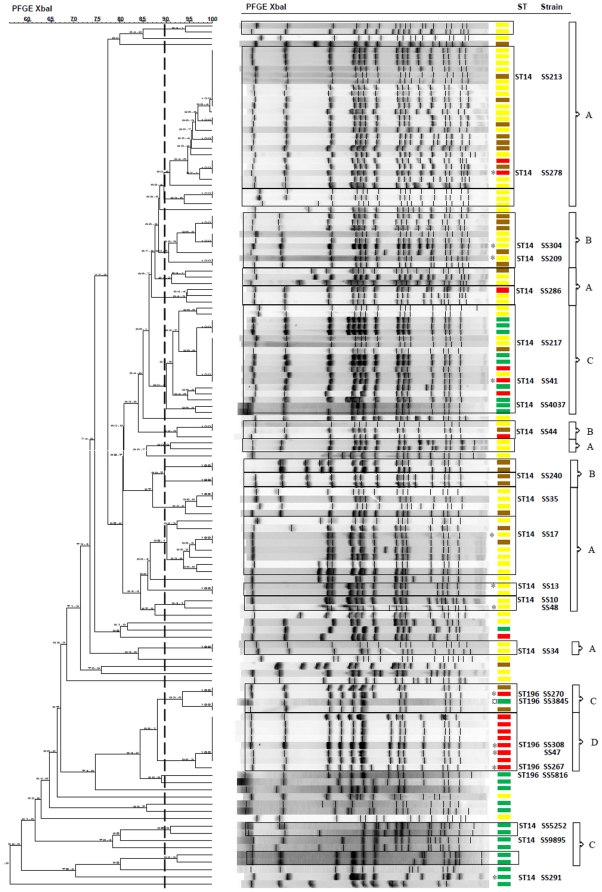
PFGE and MLST analysis of *S.* Senftenberg strains. Dendrogram generated by Bionumerics software showing PFGE fingerprint of 134 isolates of *Salmonella* Senftenberg from avian and human sources. ST: MLST sequence type of 23 isolates. Broken line indicates the division into clusters corresponding to 90% similarity with the Dice coefficient. Clusters are framed in black. Fragments ranging between 28.8 kpb and 1135 kpb were included. **Yellow rectangle:** Strains isolated from hatcheries or poultry farms (<3 weeks), **brown rectancle:** strains isolated from poultry farms (3–15 weeks) or animal feed, **red rectangle:** strains isolated at the end of the rearing period (>15 weeks) and **green rectangle:** human strains. A, B, C, D: groups of clusters. *Strains selected for the chicken experiments. **¤** This Profile is representative of five genetically identical human strains.

PFGE typing of the 134 isolates resulted in 87 different patterns, with the majority grouped into 20 clusters (≥90% similarity). For the entire panel, the Simpson index of diversity was 0.99 showing the high diversity of the strains discriminated using XbaI. The 20 clusters corresponded to four groups according to the origin of the strains (Figure 1). Eleven clusters (group A) consisted mainly of avian strains isolated from hatcheries or at the beginning of the rearing period. One cluster (group D) consisted exclusively of avian strains isolated at the end of the rearing period. The PFGE analysis also enabled human isolates to be separated. Half of these were in the same cluster and were genetically close to avian strains (group C), but the other human isolates were grouped in genetically distant clusters (<67% of similarity) (group C). The other clusters consisted of strains of different origins (hatchery and poultry farm) (group B).

The MLST analysis of the 23 isolates revealed that five isolates (SS308, SS267, SS270, SS5816 and SS3845) exhibiting genetically close PFGE patterns, belong to the same sequence type (ST196). These five isolates show more than 84% of similarity, and are genetically distant from the other strains (67% of similarity). All the other strains tested, which belong to genetically distant PFGE clusters, share the same ST14 sequence type (figure 1).

### Cecal contamination level of *S.* Senftenberg strains

**Figure 2:**
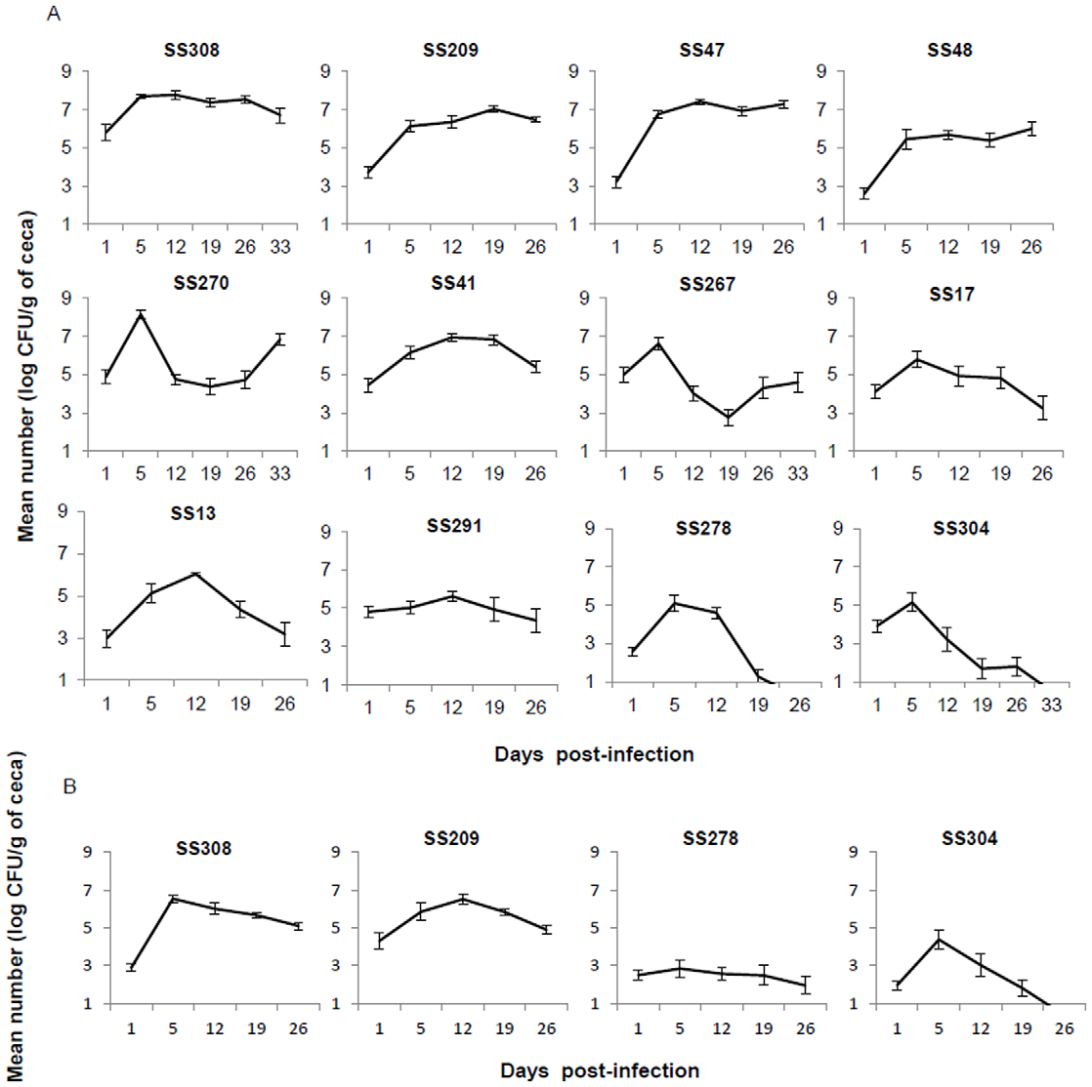
Cecal persistence and colonization levels of *S.* Senftenberg isolates in infected chicks. Number of CFU of *Salmonella* Senftenberg rifampicin-resistant strains per gram of cecum of chicks orally infected with 10^5^ CFU. (A) Enumeration was performed up to 33 days post-infection for strains SS304, SS308, SS270, SS267, and 26 days p.i for strains SS17, SS291, SS209, SS13, SS48, SS47, SS278, SS41. (B) Cecal load of chicks inoculated with four selected strains (SS308, SS209, SS304, SS278) in a second experiment up to 26 days p.i. The data presented correspond to the mean of positive and negative cecum samples ± SEM.

**Figure 3:**
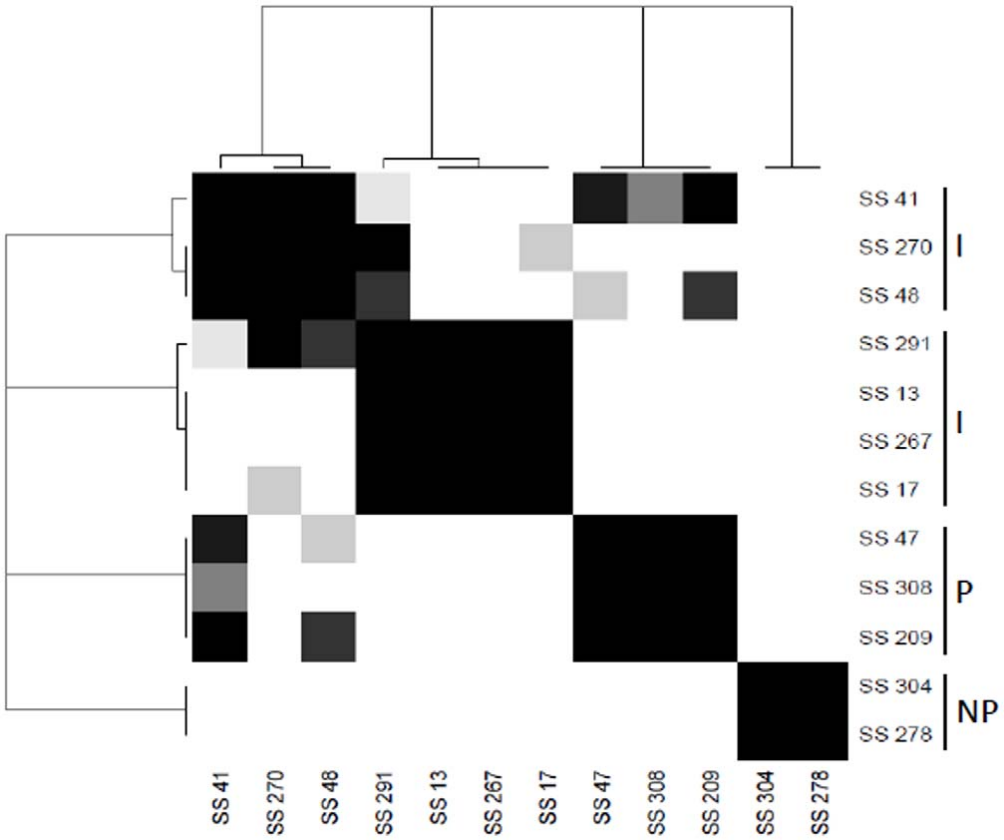
Statistical analysis comparing the cecal colonization levels of *S.* Senftenberg rifampicin-resistant strains in chicks. Dendrogram and heatmap show the clustering of strains from a complete linkage as agglomerative algorithm and the distance matrix calculated by 1-p-values where p-values were obtained from comparisons of means of strain colonization levels during the period 19 to 26 or 33 days p.i and adjusted using Tukey's method for multiple comparisons. Strains belonging to the same black square are similar and significantly different from the other strains. I: intermediate strains, P: persistent strains, NP: non-persistent strains.

On the basis of the genetic relationship between the isolates and their origin (hatchery or poultry farm), 12 rifampicin-resistant strains were selected to study their ability to colonize and persist in broiler chickens: six strains isolated at the end of the rearing period from groups A (SS278), C (SS41, SS270) and D (SS308, SS47, SS267); five strains from hatchery or the beginning of the rearing period from groups B (SS304, SS209) and A (SS17, SS13, SS48), and one human strain (SS291) (figure 2). The heatmap and dendrogram based on the distance matrix calculated by 1 - p-values and a complete linkage as agglomerative algorithm allowed the identification of highly persistent, intermediate and non-persistent strains (figure 3). The three strains identified as being persistent in this study (SS308, SS47, SS209) were able to colonize ceca of chicks and persist at high levels until the end of the experiment (6 to 7 log CFU/g) (Figure 2A). The number of bacteria increased significantly from day 1 to day 5 p.i (*P*≤0.05), remaining high and almost stable from day 5 to day 26 or 33 p.i. For the two strains identified as non-persistent (SS304, SS278), a peak value of about 5 log_10_ CFU/g was observed on day 5 p.i, but the number of bacteria in the ceca significantly decreased to values under 0.5 log CFU/g 26 days p.i. The intermediate strains are significantly different than persistent and non persistent strains (figure 3). Among the intermediate strains, two clusters can be observed. Strains SS270, SS48 and SS41 persisted and colonized the ceca for four to five weeks post infection (>5 log CFU/g) but at significantly lower levels than the persistent strains. For strains SS13, SS17, SS291 and SS267 the number of bacteria decreased after 12 days post-infection but at colonization levels which were significantly higher than those of the non persistent strains (∼3 to 4 log CFU/g).

Persistent (SS308, SS209) and non-persistent (SS304 and SS278) strains were selected for a second trial to confirm their cecal contamination level and to analyze further their virulence potential. The results (Figure 2B) confirmed the ability of strains to persist or not in chicks and confirmed that different *S.* Senftenberg strains exhibited different levels of colonization and persistence in poultry.

### Spleen colonization level of *S.* Senftenberg strains

**Figure 4:**
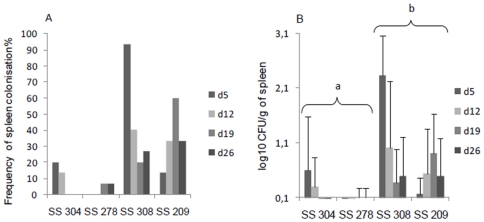
Spleen colonization of chicks infected with *S.* Senftenberg. Broiler chikens were orally infected with 10^5^ CFU. A) Frequency of spleen colonization by persistent (SS308, SS209) and non-persistent (SS278, SS304) strains of *S.* Senftenberg at different times post-infection. B) Number of CFU of *S.* Senftenberg per gram of spleen. Spleen samples which were positive after enrichment were given a value of 33 CFU. These results correspond to the mean of 15 animals (positives and negatives) ± SEM.* Letters (a–b) indicate a significant difference in means of spleen colonization levels between the groups of persistent (SS308, SS209) and non-persistent strains (SS304, SS278) at the 0.05 level.

To determine whether *S.* Senftenberg strains are able to induce systemic infection, we studied their ability to colonize the spleen of chickens. For the two persistent strains we noted a spike in spleen colonization frequency at day 5 p.i (90%) and day 19 p.i (60%) respectively, but with low colonization levels (<2.5 log_10_ CFU/g). The spleens of chickens infected with the non-persistent strains were only slightly colonized, and less than 20% of samples were positive after enrichment (Figure 4). An analysis of variance shows that all the effects (strain, day, and the interaction day*strain) are significant (p<0.05). In this condition, a pairwise comparison showed that only few significant differences were however observed between the strains to a particular day, probably due to high variability between animals. The test of the difference between the group of persistent (SS308, SS209) and non-persistent strains (SS304, SS278) was significant at level 0.05. These differences between persistent and non-persistent strains suggest that translocation of the intestinal barrier was greater for persistent strains. However, all the *S.* Senftenberg strains tested showed a poor capacity to invade the spleen compared to invasive serotypes such as *S.* Enteritidis for which the spleen colonization level is usually superior to 4 log CFU/g [26], [27].

### Antibody response induced by *S.* Senftenberg strains

To investigate whether *S.* Senftenberg is detected as a pathogen by chicks, serum samples from infected and healthy animals were collected every week to test for immune response markers (IgM and IgY) using an ELISA assay. The healthy *Salmonella*-free animals were slightly positive for IgM and IgY antibodies. Consequently, the mean antibody titers of these control animals were subtracted from those of infected animals. Our results show that the IgM antibody response induced by *S.* Senftenberg strains was very low (OD = 0.01 at 5 days pi to less than 0.2 at 26 days pi) compared to that of chicks infected by *S.* Gallinarum (OD = 1.7), an invasive serotype responsible for systemic infection [28]. The lack of specific IgY response against *S.* Senftenberg strains (DO<0.05) suggests that the very low IgM response observed in infected chickens is not specific to *S.* Senftenberg strains.

### Effect of persistent and non-persistent *S.* Senftenberg strains on intestinal morphology

To assess the effect of *S.* Senftenberg strains on the intestinal barrier, the height of cecal villi and depth of crypts of chickens infected with persistent SS308 and non-persistent SS304 strains were measured (table 1). At five days p.i, the villus height and crypt depth of infected birds were not significantly different from each other but were significantly lower than those of healthy animals (*P*≤0.05), suggesting an alteration of intestinal mucosa in all infected birds. No significant difference in villus height and crypt depth was observed between infected chickens at 26 days p.i. However, the villus height of SS304 infected birds was significantly lower than that of the control group (*P*≤0.05).

**Table 1:** Measurement of proximal cecal villus height and crypt depth of control chicks and animals infected with persistent SS308 and non-persistent SS304 strains of *S.* Senftenberg at 5 and 26 days post-infection (infection at 1 day of age).

	Parameters	Healthy animals	SS308	SS304
**5 days p.i**	**Villus height (µm)**	498±46^a^	404±63^b^	390±22^b^
	**Crypt depth (µm)**	98±6^a^	84±7b^b^	90±9^b^
**26 days p.i**	**Villus height (µm)**	678±48^a^	634±112^ab^	518±42^b^
	**Crypt depth (µm)**	128±12^b^	118±6^b^	110±9^b^

a,b The mean of proximal cecal villus heights and crypt depths ±SD. Different letters in the same line indicate a significant difference with the Mann -Whitney test (P≤0.05).

### The in vitro virulence of *S.* Senftenberg strains

**Figure 5:**
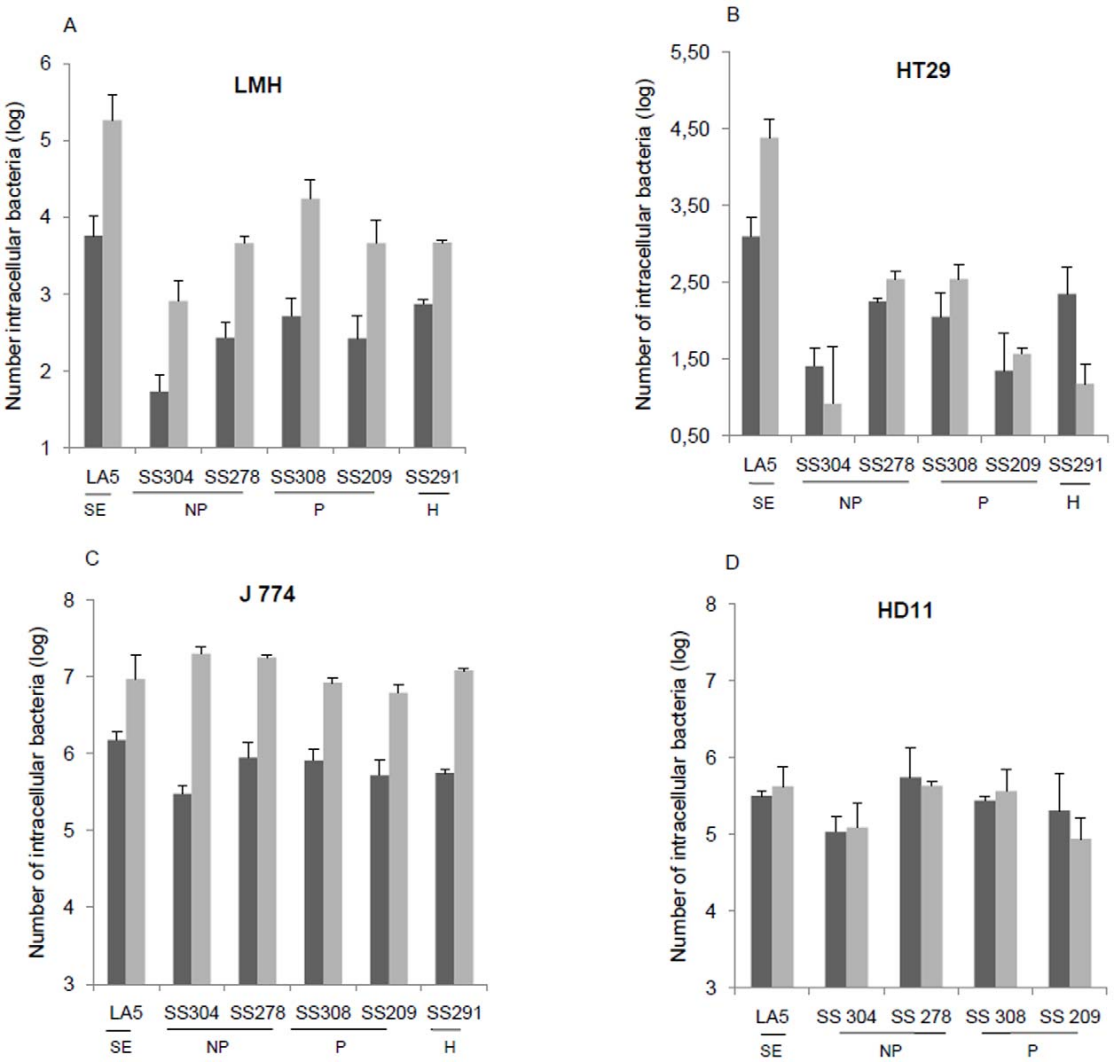
Cell invasion capacity of *S.* Senftenberg strains. In vitro invasion and intracellular multiplication were performed in epithelial cell lines (HT29 and LMH) and macrophages (HD11 and J774). Persistent (P) (SS308, SS209) and non-Persistent (NP) (SS304, SS278) strains were analyzed in addition to a human isolate (H) (SS291). Cells were infected at a MOI of 10 CFU/cell and the number of intracellular bacteria was determined 2 h (**black**) and 20 h (**grey**) post-infection. Data representing the mean ± SD of at least two independent experiments carried out in duplicate. The invasive strain of *Salmonella* Enteriditis LA5 was used as a positive control.

To investigate whether the low level of spleen colonization of infected chickens was related to poor ability to infect enterocytes or to destruction by spleen macrophages, the invasiveness of *S.* Senftenberg strains was tested in vitro. We first demonstrated that the selection of rifampicin-resistant mutants did not impact on the invasiveness capability of *S.* Senftenberg strains because no difference in adhesion, entry and intracellular levels were observed with LMH cells, except for the wild-type SS304 strain which showed a slightly higher multiplication level (∼1 log) than the SS304 rifampicin-mutant strain (data not shown). Our results show that all *S.* Senftenberg rifampicin-resistant strains were 10- to 100-fold less invasive than *S.* Enteritidis LA5 strain in both avian and human epithelial cells (LMH and HT29). This was not related to poor cell adhesion capacity, because no significant differences were observed between strains (data not shown). With LMH cells (Figure 5A), all *S.* Senftenberg strains had almost the same invasion and multiplication level, except for one non-persistent strain SS304 which appeared less invasive. With HT29 cells, the non-persistent SS304 and persistent SS209 strains had a lower invasion level than that of the other strains, but this was unrelated to their persistence phenotype. In contrast to *S.* Enteritidis, the number of intracellular bacteria for all *S.* Senftenberg strains at 20 h p.i decreased or remained stable in HT29 cells (Figure 5B). In both avian HD11 and murine J774 macrophages, the behavior of *S.* Senftenberg strains was very similar to that of *S.* Enteritidis. All these strains were able to invade macrophages at high levels and to survive in LPS-activated macrophages (Figure 5C–D).

### Virulence of *S.* Senftenberg strains in a murine model

**Figure 6:**
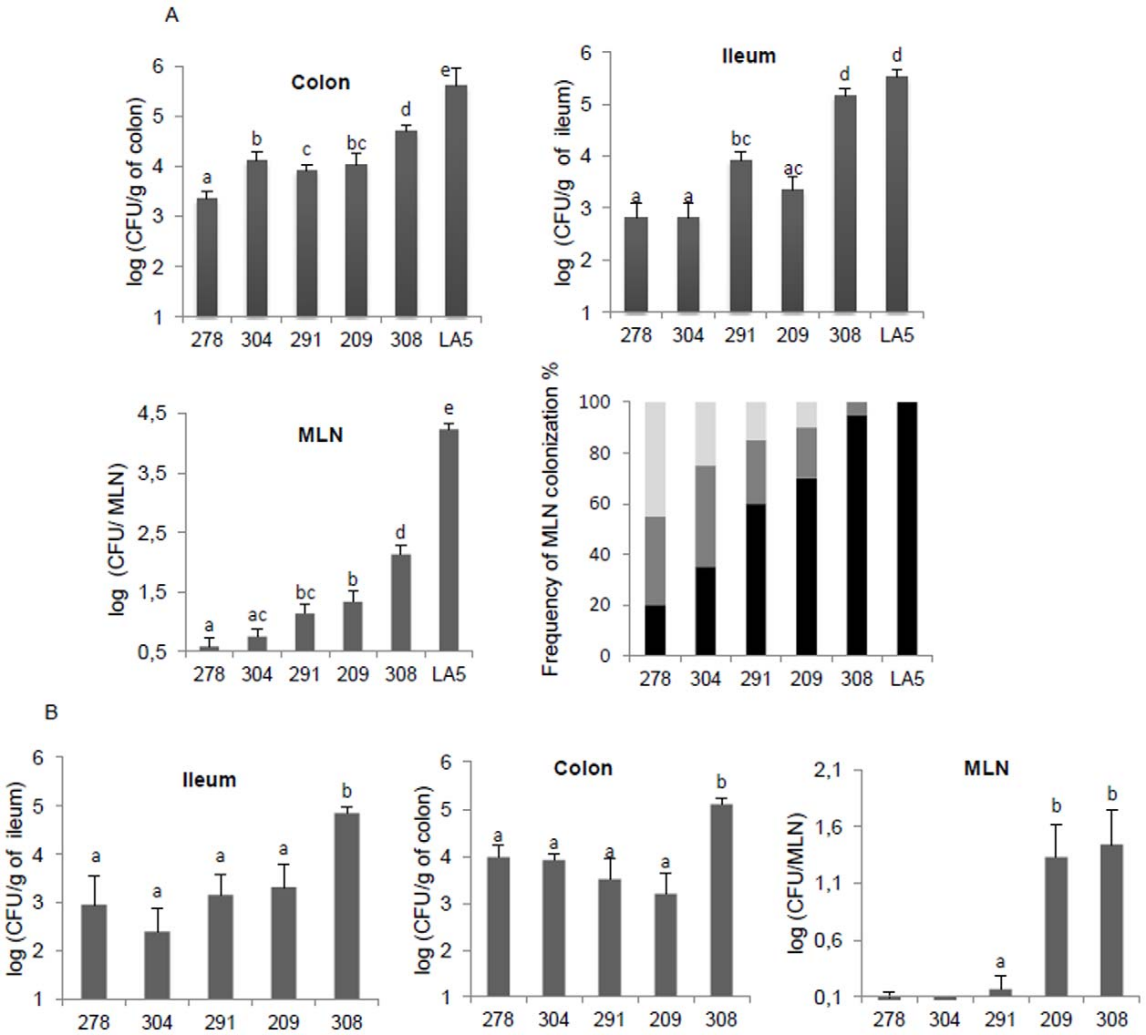
Mouse infection by S. Senftenberg strains. Groups of BALB/c mice were orally infected with 5×10^8^ bacteria of persistent (SS308, SS209), non-persistent (SS304, SS278) and human (SS291) *S.* Senftenberg strains (n = 10 for each group). (A) At day 3 p.i., the number of bacteria detected in the ileum, colon and mesenteric lymph nodes (MLN) was determined. The *S.* Enteritidis LA5 strain was used as a positive control (n = 6). The results of two independent experiments were pooled for analysis. The frequency of MLN colonization by *Salmonella* strains was calculated after direct detection (**black**) or enrichment (**grey**). **White** histogram corresponds to negative MLN samples. (B) Number of bacteria in the same organs determined 28 days p.i. Data correspond to the mean of positive and negative animals ± SEM of two independent experiments. Letters (a–e) indicate a significant difference between the different groups of infected mice (*P*<0.05).

The experiments performed on chicks showed that *S.* Senftenberg, like *S.* Enteritidis, induced an asymptomatic carrier state. However, in the mouse, *S.* Enteritidis induces a typhoid-like disease. To assess the pathogenicity of *S.* Senftenberg in a murine model, BALB/c mice were orally inoculated with persistent (SS308, SS209), non-persistent (SS278, SS304) or a human isolate (SS291). At three days p.i, intestinal colonization was 2 to 3 log_10_ less than with *S.* Enteritidis (*P*<0.05). By contrast, the level of infection of the ileum by the persistent strain SS308 was similar to *S.* Enteritidis. In the MLN, there was a significant difference in the colonization level between *S.* Enteritidis and *S.* Senftenberg strains. Non-persistent strains were found at lower frequencies and significantly lower levels than persistent strains (*P*<0.05). The human isolate SS291 showed an intermediate level of colonization between that of the persistent and non-persistent strains (Figure 6A). Unlike the LA5 strain, which is able to infect the spleen at a high level (>4 log_10_), no viable bacteria were recovered in the spleen of mice, except those of two animals infected with the persistent strain SS308 and detected only after enrichment (data not shown). This result is consistent with the inability of *S.* Senftenberg to establish a lethal systemic infection. Mice infected with *S.* Senftenberg showed no signs at any time during infection, whereas *S.* Enteritidis inoculated mice died between 7 and 11 days p.i. At 28 days p.i, all *S.* Senftenberg strains persisted in the ileum and colon. However, the persistent strain SS308 showed a higher ileum and colon colonization level, and only persistent strains were able to persist in the MLN (Figure 6B). No viable *S.* Senftenberg strains were detected in the spleen.

To confirm that the selection of rifampicin-resistant mutants was not responsible for the lack of systemic infection of *S.* Senftenberg strains, the spleen colonization levels of mice orally infected with the mutant or wild-type strains were compared. Our results showed no significant differences between the wild-type and rifampicin-resistant strains, none of the strains were able to colonize the spleen of mice (data not shown).

Overall, these results show a poor ability of *S.* Senftenberg to induce systemic infection compared to other serotypes such as *S.* Enteritidis and suggest that persistent strains have a greater ability to colonize and persist in animal tissues than non-persistent strains.

## Discussion

In poultry production, *S.* Senftenberg is generally associated with the hatchery environment, but it has recently become more frequent in animals and on poultry farms. Furthermore, some strains have been implicated in severe human infections throughout the world [29], [30].

In this study, experimental infection demonstrated that the cecal contamination capacity of chickens varied according to the *S.* Senftenberg strain involved. We identified persistent strains able to persist and colonize ceca at high levels for five weeks, and non-persistent strains which colonized ceca at lower levels and were practically eliminated five weeks p.i. The existence of distinct persistent phenotypes within *S.* Senftenberg could explain the recent emergence of this serotype in poultry flocks.

The genetic analysis of the 134 *S.* Senftenberg isolates shows that strains isolated at the end of the rearing period (superior to 15 weeks) are distributed on nearly all branches of the dendrogram and thus belong to different PFGE clusters. Moreover, the two persistent strains identified by the *in vivo* infections were distant from each others by PFGE analysis (66.3% of similarity) and have two different MLST sequence type. This result suggests that the persistence trait has been acquired independently in non-genetically related strains. However, it is interesting to note that the strains belonging to the MLST sequence type ST196 are grouped within two closely related PFGE clusters and correspond to avian strains isolated at the end of the rearing period (group D) or to human isolates (group C). Moreover two out of three of the persistent strains in chickens (SS308, SS47) belong to these PFGE clusters suggesting that ST196 could be used to identify some persistent strains. Similarly, PFGE analysis also allowed human isolates to be separated, half of which were in the same cluster while the others were in genetically distant clusters.

Among the 12 isolates tested *in vivo*, only two were identified as non-persistent. One was the SS304 strain from a cluster of group B and originated from hatchery, which is in line with its non-persistence in our chicken experimental infection. The other strain SS278 was isolated after 24 weeks of rearing. This apparently contradictory finding could be explained by external contamination or persistence in the environment. This hypothesis is supported by the fact that the SS278 strain belongs to PFGE cluster common to group A strains. Moreover, no checks were carried out during the rearing period to test for isolates originating from hatcheries or one-day-old chicks, thus explaining the persistence of some of these isolates in animals. That was the case of the two strains SS209 and SS48 isolated from one-day-old animal feces and hatchery respectively which were able to colonize and persist in chickens experimentally infected.

Although we have shown that persistent *S.* Senftenberg strains are able to induce an asymptomatic carrier state in chicks, in a similar way to *S.* Enteritidis, the hallmark of *S.* Senftenberg is its poor capacity to induce systemic infection in either chicks or mice. *S.* Senftenberg strains induced fewer morphological changes in the intestine of chicks than described for *S.* Enteritidis during the first days of infection [31], colonized the spleen at a low level, and consequently did not induce an antibody response. Similarly, in the mouse model, which is the gold standard for assessing *Salmonella* virulence [32], there was a high level of intestinal colonization but no spleen colonization, which can in part be explained by the poor ability to infect MLN. One hypothesis is that *S.* Senftenberg is unable to survive in macrophages and is thus eliminated before reaching the internal organs [33]. However, our in vitro results, showing that *S.* Senftenberg strains were able to invade and survive in activated macrophages at the same levels as *S.* Enteritidis, suggest that this hypothesis is not valid. Another possible explanation is that the low virulence of *S.* Senftenberg is related to its low capacity to translocate the intestinal barrier. This hypothesis is supported by the observation that all *S.* Senftenberg strains were less invasive for avian and human epithelial cells than *S.* Enteritidis and were unable to multiply within human enterocytes, unlike *S.* Enteritidis. This is probably not related to an absence of the *Salmonella* pathogenicity island-1 (SPI-1), as previously suggested [34], because the *invA* and *hilA* virulence genes were detected in all *S.* Senftenberg strains used in our study (data not shown).

When comparing persistent and non-persistent strains, it can be seen that they vary in their capacity to colonize and persist in animal tissues. However, persistence of *S.* Senftenberg strains is not correlated with the invasion and multiplication levels in epithelial cells and activated macrophages. These findings suggest that the difference of colonization levels in chicken spleen and mice MLN between strains could be attributed to the difference in the bacterial load in intestinal contents. Nevertheless, the greater persistence in the MLN of persistent strains does not support this hypothesis and suggests that non-persistent strains are also impaired in their ability to resist the innate immune response.

In our study, we also showed that the human strain SS291 colonized the ceca of chicks and the intestinal tract and MLN of mice at levels between those of persistent and non-persistent strains. Moreover, like all the *S.* Senftenberg strains tested, the human strain did not induce systemic disease in mice. One hypothesis is that the infected humans had some other factors that facilitated the infection process. Another hypothesis is that the systemic infection model of the mouse is not suitable for testing the virulence of *S.* Senftenberg in humans. This later hypothesis is supported by the data obtained from a food-borne disease outbreak by Hu *et al.* who identified several *S.* Senftenberg clinical isolates from immunocompetent patients infected with no other pathogens [34]. In line with our results, they showed that *S.* Senftenberg isolates from humans could not induce an intestinal pathology or systemic infection in the mouse model of enterocolitis. Overall, we consider that all *S.* Senftenberg strains are potentially pathogenic for humans; persistent strains would thus present a greater risk for human health than non-persistent strains, due to their ability to persist and colonize the intestine, while non-persistent strains would be eliminated before slaughter and food processing.

Human clinical cases caused by the Senftenberg serotype are not necessarily linked to avian sources. In fact, the results of our PFGE analysis show that although numerous human isolates are genetically close to avian isolates, others are grouped in distant clusters suggesting a divergent evolution. We could thus suppose that these human isolates are mainly related to sources other than poultry or the area of contamination (e.g., travel-acquired infection). This hypothesis is supported by recent outbreaks of infection of non-avian origins, such as the one linked to pre-packed basil [35]. This serotype can adhere to plant leaves [36] and is also able to survive in a hypersaline environment, and it is thus one of the main serotypes associated with seawater and seafood products [37], [38]. Further research to study the virulence and persistence capacity of these human strains of different origins would be of interest.

To conclude, the increased presence of *S.* Senftenberg on poultry farms could be explained by differing capacities of the persistent and non-persistent strains to colonize and survive in chick and mouse tissues. Persistent strains could also be more able to survive in the intestinal content of chicken. The role played by these factors requires further investigation.

## References

[1] Voetsch AC, Van Gilder TJ, Angulo FJ, Farley MM, Shallow S (2004). FoodNet estimate of the burden of illness caused by nontyphoidal *Salmonella* infections in the United States.. Clin Infect Dis.

[2] Rabsch W, Tschape H, Baumler AJ (2001). Non-typhoidal salmonellosis: emerging problems.. Microbes Infect.

[3] Velge P, Cloeckaert A, Barrow P (2005). Emergence of Salmonella epidemics: the problems related to Salmonella enterica serotype Enteritidis and multiple antibiotic resistance in other major serotypes.. Vet Res.

[4] Anonymous (2010). Analysis of the baseline survey on the prevalence of *Campylobacter* in broiler batches and of *Campylobacter* and *Salmonella* on broiler carcasses in the EU, 2008 - Part A: *Campylobacter* and *Salmonella* prevalence estimates.. The EFSA journal.

[5] Gal-Mor O, Valinsky L, Weinberger M, Guy S, Jaffe J (2010). Multidrug-resistant *Salmonella enterica* serovar Infantis, Israel.. Emerg Infect Dis.

[6] Hamada S, Hashimoto H, Tasaka T, Tsuchiya Y (1959). Studies on chick Salmonellosis: II. *Salmonella* Senftenberg infection in chicks.. Japanese Journal of Veterinary Research.

[7] Hume ME, Kubena LF, Edrington TS, Donskey CJ, Moore RW (2003). Poultry digestive microflora biodiversity as indicated by denaturing gradient gel electrophoresis.. Poult Sci.

[8] Liebana E, Guns D, Garcia-Migura L, Woodward MJ, Clifton-Hadley FA (2001). Molecular typing of *Salmonella* serotypes prevalent in animals in England: assessment of methodology.. J Clin Microbiol.

[9] Anonymous (2007). Report of the Task Force on Zoonoses Data Collection on the Analysis of the baseline survey on the prevalence of *Salmonella* in holdings of broiler flocks of Gallus gallus-Part B: factors related to *Salmonella* flock prevalence, distribution of *Salmonella* serovars, and antimicrobial resistance patterns.. The EFSA journal.

[10] Pedersen TB, Olsen JE, Bisgaard M (2008). Persistence of *Salmonella* Senftenberg in poultry production environments and investigation of its resistance to desiccation.. Avian Pathol.

[11] Kay RS, Vandevelde AG, Fiorella PD, Crouse R, Blackmore C (2007). Outbreak of healthcare-associated infection and colonization with multidrug-resistant *Salmonella* enterica serovar Senftenberg in Florida.. Infect Control Hosp Epidemiol.

[12] L'Ecuyer PB, Diego J, Murphy D, Trovillion E, Jones M (1996). Nosocomial outbreak of gastroenteritis due to *Salmonella* Senftenberg.. Clin Infect Dis.

[13] Allen-Vercoe E, Dibb-Fuller M, Thorns CJ, Woodward MJ (1997). SEF17 fimbriae are essential for the convoluted colonial morphology of *Salmonella* Enteritidis.. FEMS Microbiol Lett.

[14] Ribot EM, Fair MA, Gautom R, Cameron DN, Hunter SB (2006). Standardization of pulsed-field gel electrophoresis protocols for the subtyping of Escherichia coli O157:H7, *Salmonella*, and *Shigella* for PulseNet.. Foodborne Pathog Dis.

[15] Kerouanton A, Marault M, Lailler R, Weill FX, Feurer C (2007). Pulsed-field gel electrophoresis subtyping database for foodborne *Salmonella enterica* serotype discrimination.. Foodborne Pathog Dis.

[16] Hunter PR, Gaston MA (1988). Numerical index of the discriminatory ability of typing systems: an application of Simpson's index of diversity.. J Clin Microbiol.

[17] Kidgell C, Reichard U, Wain J, Linz B, Torpdahl M (2002). *Salmonella* typhi, the causative agent of typhoid fever, is approximately 50,000 years old.. Infect Genet Evol.

[18] Pardon P, Popoff MY, Coynault C, Marly J, Miras I (1986). Virulence-associated plasmids of Salmonella serotype Typhimurium in experimental murine infection.. Ann Inst Pasteur Microbiol.

[19] Goodlad RA, Levi S, Lee CY, Mandir N, Hodgson H (1991). Morphometry and cell proliferation in endoscopic biopsies: evaluation of a technique.. Gastroenterology.

[20] Williams J, Mallet S, Leconte M, Lessire M, Gabriel I (2008). The effects of fructo-oligosaccharides or whole wheat on the performance and digestive tract of broiler chickens.. Br Poult Sci.

[21] Skov MN, Feld NC, Carstensen B, Madsen M (2002). The serologic response to *Salmonella* Enteritidis and *Salmonella* Typhimurium in experimentally infected chickens, followed by an indirect lipopolysaccharide enzyme-linked immunosorbent assay and bacteriologic examinations through a one-year period.. Avian Dis.

[22] Kawaguchi T, Nomura K, Hirayama Y, Kitagawa T (1987). Establishment and characterization of a chicken hepatocellular carcinoma cell line, LMH.. Cancer Res.

[23] Temoin S, Roche SM, Grepinet O, Fardini Y, Velge P (2008). Multiple point mutations in virulence genes explain the low virulence of *Listeria Monocytogenes* field strains.. Microbiology.

[24] Amy M, Velge P, Senocq D, Bottreau E, Mompart F (2004). Identification of a new *Salmonella enterica* serovar Enteritidis locus involved in cell invasion and in the colonisation of chicks.. Res Microbiol.

[25] Roche SM, Gracieux P, Milohanic E, Albert I, Virlogeux-Payant I (2005). Investigation of specific substitutions in virulence genes characterizing phenotypic groups of low-virulence field strains of *Listeria Monocytogenes*.. Appl Environ Microbiol.

[26] Sadeyen JR, Trotereau J, Velge P, Marly J, Beaumont C (2004). *Salmonella* carrier state in chicken: comparison of expression of immune response genes between susceptible and resistant animals.. Microbes Infect.

[27] Van Immerseel F, De Buck J, De Smet I, Mast J, Haesebrouck F (2002). Dynamics of immune cell infiltration in the caecal lamina propria of chickens after neonatal infection with a *Salmonella* Enteritidis strain.. Dev Comp Immunol.

[28] Chadfield MS, Brown DJ, Aabo S, Christensen JP, Olsen JE (2003). Comparison of intestinal invasion and macrophage response of *Salmonella* Gallinarum and other host-adapted *Salmonella enterica* serovars in the avian host.. Vet Microbiol.

[29] Pezzoli L, Elson R, Little C, Fisher I, Yip H (2007). International outbreak of *Salmonella* Senftenberg in 2007.. Euro Surveill.

[30] Rushdy AA, Stuart JM, Ward LR, Bruce J, Threlfall EJ (1998). National outbreak of *Salmonella* senftenberg associated with infant food.. Epidemiol Infect.

[31] Borsoi A, Santos LRd, Rodrigues LB, Moraes HLdS, Salle CTP (2011). Behavior of *Salmonella* heidelberg and *Salmonella* Enteritidis strains following broiler chick inoculation: evaluation of cecal morphometry, liver and cecum bacterial counts and fecal excretion patterns.. Brazilian Journal of Microbiology.

[32] Eisenstein TK, Killar LM, Sultzer BM (1984). Immunity to infection with *Salmonella* Typhimurium: mouse-strain differences in vaccine- and serum-mediated protection.. J Infect Dis.

[33] Withanage GS, Mastroeni P, Brooks HJ, Maskell DJ, McConnell I (2005). Oxidative and nitrosative responses of the chicken macrophage cell line MQ-NCSU to experimental *Salmonella* infection.. Br Poult Sci.

[34] Hu Q, Coburn B, Deng W, Li Y, Shi X (2008). *Salmonella enterica* serovar Senftenberg human clinical isolates lacking SPI-1.. J Clin Microbiol.

[35] Pezzoli L, Elson R, Little CL, Yip H, Fisher I (2008). Packed with *Salmonella*-investigation of an international outbreak of *Salmonella* Senftenberg infection linked to contamination of prepacked basil in 2007.. Foodborne Pathog Dis.

[36] Berger CN, Shaw RK, Brown DJ, Mather H, Clare S (2009). Interaction of *Salmonella* enterica with basil and other salad leaves.. ISME J.

[37] Martinez-Urtaza J, Peiteado J, Lozano-Leon A, Garcia-Martin O (2004). Detection of *Salmonella* Senftenberg associated with high saline environments in mussel processing facilities.. J Food Prot.

[38] Martinez-Urtaza J, Saco M, Hernandez-Cordova G, Lozano A, Garcia-Martin O (2003). Identification of *Salmonella* serovars isolated from live molluscan shellfish and their significance in the marine environment.. J Food Prot.

